# Supplementary material for: Modeling glioblastoma heterogeneity as a dynamic network of cell states
Source: Mol Syst Biol. 2021 Sep 16;17(9):e10105. doi: 10.15252/msb.202010105 (PMC8444284; doi:10.15252/msb.202010105)
Supplement: Supplementary file 5 — Source Data for Figure 3 [file MSB-17-e10105-s001.zip › Figure3A_sourcedata/GSEA_3065/nosigdb_state6.GseaPreranked.1622718443993/DOROSZKO_U3180MG_CLUSTER_6.html]

Details for gene set DOROSZKO\_U3180MG\_CLUSTER\_6[GSEA]

|  || Dataset | state6 |
| Phenotype | NoPhenotypeAvailable |
| Upregulated in class | na\_pos |
| GeneSet | DOROSZKO\_U3180MG\_CLUSTER\_6 |
| Enrichment Score (ES) | 0.40493897 |
| Normalized Enrichment Score (NES) | 1.4416516 |
| Nominal p-value | 0.054669704 |
| FDR q-value | 0.072295524 |
| FWER p-Value | 0.827 |
Table: GSEA Results Summary

  

Fig 1: Enrichment plot: DOROSZKO\_U3180MG\_CLUSTER\_6      
 Profile of the Running ES Score & Positions of GeneSet Members on the Rank Ordered List

  

| PROBE | GENE SYMBOL | GENE\_TITLE | RANK IN GENE LIST | RANK METRIC SCORE | RUNNING ES | CORE ENRICHMENT || 1 | SARS |  |  | 35 | 0.260 | 0.0866 | Yes |
| 2 | FILIP1L |  |  | 175 | 0.183 | 0.1346 | Yes |
| 3 | LARP6 |  |  | 508 | 0.122 | 0.1392 | Yes |
| 4 | CEBPG |  |  | 640 | 0.111 | 0.1630 | Yes |
| 5 | SQSTM1 |  |  | 719 | 0.104 | 0.1903 | Yes |
| 6 | ATF4 |  |  | 777 | 0.100 | 0.2187 | Yes |
| 7 | PIK3R1 |  |  | 785 | 0.100 | 0.2525 | Yes |
| 8 | ASNS |  |  | 850 | 0.094 | 0.2781 | Yes |
| 9 | SNHG12 |  |  | 865 | 0.093 | 0.3090 | Yes |
| 10 | GDF15 |  |  | 888 | 0.092 | 0.3384 | Yes |
| 11 | CEBPB |  |  | 943 | 0.089 | 0.3632 | Yes |
| 12 | TARS |  |  | 1183 | 0.076 | 0.3624 | Yes |
| 13 | SESN2 |  |  | 1271 | 0.073 | 0.3779 | Yes |
| 14 | YARS |  |  | 1337 | 0.070 | 0.3947 | Yes |
| 15 | WDR45B |  |  | 1449 | 0.066 | 0.4049 | Yes |
| 16 | CLIC4 |  |  | 2035 | 0.047 | 0.3544 | No |
| 17 | INSM1 |  |  | 2051 | 0.046 | 0.3688 | No |
| 18 | GNG12 |  |  | 2317 | 0.038 | 0.3518 | No |
| 19 | PAK1 |  |  | 2589 | 0.031 | 0.3317 | No |
| 20 | CASP3 |  |  | 2634 | 0.030 | 0.3373 | No |
| 21 | TMOD1 |  |  | 2745 | 0.028 | 0.3344 | No |
| 22 | MAP1B |  |  | 2893 | 0.024 | 0.3261 | No |
| 23 | XPOT |  |  | 2982 | 0.022 | 0.3238 | No |
| 24 | LYST |  |  | 3444 | 0.012 | 0.2754 | No |
| 25 | FBXO32 |  |  | 3674 | 0.007 | 0.2518 | No |
| 26 | CXADR |  |  | 4321 | -0.005 | 0.1799 | No |
| 27 | IQGAP2 |  |  | 5055 | -0.019 | 0.1030 | No |
| 28 | SMAD1 |  |  | 5360 | -0.026 | 0.0772 | No |
| 29 | PDLIM1 |  |  | 5379 | -0.026 | 0.0841 | No |
| 30 | ARID5A |  |  | 5559 | -0.030 | 0.0740 | No |
| 31 | MEF2A |  |  | 6220 | -0.043 | 0.0136 | No |
| 32 | UBE3A |  |  | 6770 | -0.058 | -0.0289 | No |
| 33 | CNKSR3 |  |  | 7470 | -0.080 | -0.0808 | No |
| 34 | WLS |  |  | 7516 | -0.082 | -0.0573 | No |
| 35 | AC027031.2 |  |  | 7557 | -0.084 | -0.0326 | No |
| 36 | LNPEP |  |  | 7742 | -0.093 | -0.0213 | No |
| 37 | IARS |  |  | 8179 | -0.120 | -0.0293 | No |
| 38 | PGM2L1 |  |  | 8304 | -0.132 | 0.0027 | No |
| 39 | TAGLN2 |  |  | 8457 | -0.153 | 0.0387 | No |
Table: GSEA details [plain text format]

  

Fig 2: DOROSZKO\_U3180MG\_CLUSTER\_6: Random ES distribution      
 Gene set null distribution of ES for **DOROSZKO\_U3180MG\_CLUSTER\_6**
